# Prevalence of Depression and Associated Factors Among Adult Eritreans in Norway

**DOI:** 10.1177/00207640251384523

**Published:** 2025-11-03

**Authors:** Fesehatsion Afewerki Haylom, Sudan Prasad Neupane

**Affiliations:** 1 Department of Community Medicine and Global Health, Institute of Health and Society, Faculty of Medicine, University of Oslo, Norway; 2 National Centre for Suicide Research and Prevention, Institute of Clinical Medicine, Faculty of Medicine, University of Oslo, Norway; 3 Oral Health Centre of Expertise in Rogaland, Stavanger, Norway

**Keywords:** depression, immigrant, mental health, Eritreans, acculturation

## Abstract

**Background:**

The higher rates of depression among immigrant populations are often attributed to socioeconomic disadvantage, adverse life events and other pre-migration circumstances and challenges with reintegration process. We aimed to determine the prevalence of depression and examine the associated factors among Eritrean immigrants living in Oslo and surrounding areas.

**Method:**

A convenience sample of 243 adult Eritreans, 114 women and 129 men, aged 35.7 (*SD* 9.7) years were enrolled. A battery of structured instruments was administered to assess the sociodemographic, psychiatric, and acculturation-related variables, including the screening of depressive symptoms using the Center for Epidemiological studies-Depression scale (CES-D) and major depression (recent 2-weeks and 12 months) using the Major Depression Inventory (MDI). Statistical analyses were conducted using χ^2^-tests, Student’s *t*-tests, and logistic regression.

**Results:**

The prevalence rates of past week depressive symptoms, current and 12 months’ depression in the sample were 0.8%, 1.2% and 16.9% respectively. Factors associated with 12-month depression were increasing age, longer duration of stay since migration, presence of PTSD symptoms and higher number of adverse life events experienced. PTSD symptoms (OR = 4.2 (95% CI: 1.9 to 9.3, *p* < .001) and the number of adverse life events (OR = 1.05 (95% CI: 1.01 to 1.1, *p < *.001) were identified as independent correlates of 12-month depression.

**Conclusion:**

Every 6th Eritrean in our sample was found to screen positive for 12-month depression, with almost 15% participants reporting that life was not worth living. The study findings call for targeted programmes to address poor mental health among this group of immigrants.

## Introduction

Depressive disorder is a major public health issue globally. Data from the Global Burden of Disease Study showed that 280 million people suffered from a depressive disorder in 2019, a 64% increase from 1990 (GBD 2019 Mental Disorders Collaborators, 2022). In addition to diagnosed cases of depression found in 7.0% of the general population, 13.8% of women and 9.7% men experience sub-clinical symptoms of depression at any time (Zhang et al., 2023), further escalating the associated burden. Depression has a complex aetiology that involves interaction between biological, psychological and social factors, however much of the underlying mechanisms remain to be elucidated. At the individual level, factors known to contribute to depression symptoms and depressive disorders include female gender, experiences of adverse life events, lack of social support, physical ill-health, and financial difficulties (Marx et al., 2023). The relevance of social determinants of mental health are increasingly recognised and investigated, demonstrating socioeconomic disadvantage as an important risk factor for depression including in the European context (Freeman et al., 2016; Rai et al., 2013). Against this backdrop, it is important to investigate the burden and factors relevant to depression among immigrant populations.

Although many immigrants will have improved living conditions when appropriately resettled in a developed economy, more remains to be understood how dynamic interactions between the individual’s social, biological, and psychological background and current life situation may impact mental health status. In general, immigrants in Europe have higher prevalence rates of depressive symptoms compared to the native population (Missinne & Bracke, 2012). Psychological stress associated with resettlement as well as acculturation may be prominent during the early phase of immigration and may influence both younger and adult populations (Potochnick & Perreira, 2010; Sirin et al., 2013; Xu & Chi, 2013). Indeed, the risk for depression and other mental disorders will be influenced by distal factors contributing to pre-immigration vulnerability such as personal and familial history of the individual compounded by proximal factors including those inherent to the host society. For example, a multi-national study of 23 European countries estimated that up to 40% of the increased depression level among ethnic minorities was related to the experience of ethnic discrimination (Missinne & Bracke, 2012). Similarly, other background variables play a role as exemplified by an Australian study showing depression among 19.7% foreign born non-English speaking immigrants compared to the prevalence rate of 9.2% among foreign-born English-speaking immigrants (Straiton et al., 2014).

Several recent investigations have found varying levels of depression and other mental health problems among Eritrean immigrants living in Europe, Australia, and North America. An important aspect of findings from studies on immigrant populations is that initially lower rates of depression may level off and eventually reach native levels as the duration of stay lengthens (Hollander et al., 2020; Priebe et al., 2016). Such a trend was confirmed using dispensing rates of psychotropic medication as a proxy for mental health problems among refugee samples including those from Eritrea settled in Sweden (Brendler-Lindqvist et al., 2014). A review on the topic suggested similar explanations (i.e., acculturative stress, poor social support, deprived socioeconomic conditions, multiple negative life events, experiences of discrimination and traumatic pre-migration experiences) for the higher rates of mental illness among immigrants from low- and middle-income countries residing in Norway compared to the native population (Abebe et al., 2014).

The immigrant population in Europe has grown substantially during the recent decades. According to the Statistics bureau of Norway, as of mid-2023, every sixth Norwegian resident was a foreign-born immigrant and 5.1% of Norwegian residents had a refugee background (Statistics Norway, 2024). It is well known that in Europe, immigrants and refugees – especially those with non-European backgrounds – are disproportionately affected by poor mental health (Levecque & Van Rossem, 2015). As of January 1, 2025, there were 35,189 Eritrean immigrants living in Norway, including 9,666 children born to Eritrean parents (Statistics Norway, 2025b). This makes them the eighth largest immigrant group in the country. Eritrea underwent a nationalist revolution and liberation movement since 1960s until its independence from Ethiopia in 1993. This followed post-war tensions and internal repression including sustained national mobilisation and prolonged conscription, social mistrust, and economic deprivation – all of which contributed to significant youth emigration (Hepner, 2012). Most Eritrean immigrants to Norway between 1990 and 2024 arrived as asylum seekers (approximately 70%) and, according to Statistics Norway, they tend to reside more frequently in less central municipalities than other immigrant groups such as Afghani or Somalians. Furthermore, relatively high- every fourth Eritrean in Norway lives alone, and every fifth Eritrean is dependent on social welfare (Lysen et al., 2024). African immigrant population as a group has lower levels of income and education. However, considerably more Eritrean adults in Norway are engaged in employment or education (74.0%) compared to the broader population of African immigrants (62.1%) (Lysen et al., 2024; Statistics Norway, 2025c). These characteristics distinguish the Eritrean immigrant population and suggest that their integration experiences may differ from those of other immigrant groups, with potential implications for mental health outcomes. Very little mental health research has been conducted among this population. Thus, we set out to determine the prevalence and correlates of depression among Eritrean immigrants living in the capital Oslo and surrounding areas. We examined multiple pre-migration, post-migration and sociodemographic factors focusing on trauma history, major life events, alcohol/substance use, sociodemographic status as well as acculturation parameters to identify their associations with depression.

## Methods

### Study Design and Ethics

A cross-sectional study was carried out among adult Eritrean immigrants living in Oslo and surrounding areas. A conveniently recruited sample (*N* = 243) was interviewed by a single researcher using fully standardised questionnaire to assess demographic, immigration, and mental-health variables. The study protocol received ethical review and approval from the Norwegian Regional Ethics Committee (REK) Region South-East Oslo (ref # 2015/1616). Each participant signed a written-informed consent prior to enrolment.

### Study Setting and Participants

Participants were recruited through open invitation letters posted in the notice boards of public avenues frequented by our target populations at the Eritrean Shalom Pentecostal church, the Eritrean Orthodox Church and the Eritrean Women Union in Oslo. Interested individuals contacted the researcher for possible participation. Inclusion criteria for the present study were (a) an immigrant aged 18 years or above (b) self-declared identity as having Eritrean background by birth (c) possessing a Norwegian residence permit (d) migrated to Norway at least 1 year before the contact with the researcher and (e) currently residing in the municipality of Oslo or nearby locations. We included no more than one individual from the same family and excluded those receiving specialised psychiatric follow-up. Six individuals initially considered for enrollment declined to participate.

### Sociodemographic Details

Sociodemographic characteristics were assessed using a pro-forma developed for this study. It included the variables age, gender, marital status, family size, education, employment status, and religiosity.

### Immigration- and Acculturation-Related Variables

We recorded reasons for immigration as involuntary (such as for asylum) or voluntary (such as for education or employment purposes). Family situation was categorised as (living with the core family or awaiting reunification with a close family member). The length of residence in Norway was noted and presented into three categories: short-term (⩽7 years), medium-term (8–12 years), and long-term (⩾13 years), to reflect distinct phases of immigrant settlement and adaptation. The variable was treated as a continuous measure in multivariate analysis. Additional questions recorded self-reports of acculturation-related variables: whether the participant was happy living in Norway, the level of skill in Norwegian language, and whether the participant read Norwegian news. Specific items also assessed variables indicating the level of integration and perceived social support: visited by Norwegians, received help/support from Norwegians, and access to housing and employment. Finally, social engagement within the Eritrean community was assessed with the item frequency of participation in social activity.

### Psychometric Assessment

Depressive symptoms during the preceding week were assessed using the Center for Epidemiological studies-Depression scale (CES-D). Originally developed by Radloff, in 1977, CES-D is a self-report questionnaire useful for screening depressive symptoms in community as well as clinical settings. This questionnaire has been widely used, translated into multiple languages and found to show acceptable psychometric properties (Roberts, 1980; Vilagut et al., 2016). The Tigrigna version of the tool has been validated in a sample of Eritrean immigrants in the United States, and we used, upon approval, the translated and adapted version of the tool (Mogos, 2011). The instrument demonstrated strong internal consistency (Cronbach’s α = .86) and high test-retest reliability (*r* = .91). The instrument includes 20 items, each item consisting of the relevant symptoms and scored between 0 (less than a day), 1 (1–2 days), 2 (3–4 days), and 3 (5–7 days) for symptoms experienced during the last week. A sum score of 16 or more was designated as possible depression.

Major depressive disorder during the recent 2-weeks and 12-months was assessed using the Major Depression Inventory (Bech et al., 2001). The inventory includes 12 questions for each symptom and scored between 0 (at no time), 1 (some of the time), 2 (slightly less than half of the time), 3 (slightly more than half of the time), 4 (most of the time), 5 (all the time). The instrument showed an adequate internal consistency (Cronbach’s α = .89) within our sample. A cut-off score of 20 or more indicates that participants are at risk for clinical depression (Bech et al., 2001; Olsen et al., 2003). A test score less than 20 indicates no depression; 20 to 24 indicates mild depression; 25 to 29 indicates moderate depression and a score over 30 indicates severe depression. Additionally, the MDI question ‘Have you felt during the last12-months that life wasn’t worth living?’ was analysed and reported separately.

We administered the Life Events Checklist (LEC), a commonly used screening instrument to identify experience of incidents and events that may potentially cause PTSD (Gray et al., 2004). The civilian version of the PCL (PCL-C) (Weathers et al., 1994) was used to screen the participants for possible PTSD. The instrument includes 17 questions each scored from 1 to 5 depending on the symptom severity. In samples with high rates of trauma exposure, as is the case in the current study, a cut-off score of 28 has been recommended for screening possible PTSD (Lang et al., 2003). Due to this relatively low cutoff value, we operationalised the variable as PTSD symptoms present for those scoring 28 or above. The CAGE questionnaire adapted to include drug use was utilised to screen participants with alcohol and drug problems (Ewing, 1984).

Most Eritreans use Tigrigna and therefore all instruments were administered in this language. MDI, PCL-C, and the CAGE questionnaire underwent translations using the standard protocols of the World Health Organization (Artounian et al., 2012; Mogos, 2011). The first author (FAH) who has Tigrigna as his mother tongue carried out all interviews.

### Statistical Analysis

Our main analytical plan was descriptive, with data reported as frequency, percentage, mean, and standard deviation. Demographic and clinical characteristics were explanatory variables, and the main outcome measure was possible major depression during the 12-month period preceding interview according to Major Depression Inventory. Differences between categories were examined using Pearson’s χ^2^-test. Student’s *t*-test was used to examine the associations between frequency of adverse life events experiences (happened to self and witnessed events), and depression. Binary logistic regression analysis was done to examine the associations between demographic and clinical variables shown to be different across groups with and without 12-month depression. Finally, a multiple logistic regression model was developed by fitting the variables age, sex, length of stay in Norway, presence of PTSD symptoms, alcohol problems, and number of adverse life events. A fitted model provided independent effect of each variable into describing 12-month depression. Bivariate and adjusted odds ratios with corresponding 95% confidence intervals are reported. A *p* value of <.05 was considered significant. All statistical analyses were run on the IBM SPSS Statistics version 29.0.0.0.

## Results

### Sociodemographics

Of the 243 participants (mean age 35.7 ± 9.7 years), 53% were men and 47% were women; 60% were married and two-thirds (66.3%) reported of being active in some forms of employment. Only 13.2% participants cited voluntary reasons for immigration; 4% participants were waiting for family reunification. In all, 63.0% participants had been living in Norway for less than 7 years. The majority (99.6%) of the participants were Christians who attended church services regularly. All but two participants were from the ethnic group Tigrigna. Table 1 shows sociodemographic and immigration-related characteristics of the study population by gender.

**Table 1.** Sociodemographic and Immigration-Related Characteristics of the Study Population.

Characteristics	Total (*N* = 243; 100%)		Women (*n* = 114; 46.9%)		Men (*n* = 129; 53.1%)	
	*n*	%	*n*	%	*n*	%
Age in years^a^	35.7	9.7	36.4	9.0	35.2	10.2
Marital status						
Single	84	34.6	32	28.1	52	40.3
Married	145	59.7	71	62.3	74	57.4
Divorced	14	5.7	11	9.6	3	2.3
Years of education						
Up to 7 years	14	5.8	13	11.4	3	2.3
8–12 years	162	67.2	72	63.2	90	69.8
13 years or more	65	27.0	29	25.4	36	27.9
Employment						
Currently unemployed	82	33.7	40	35.1	42	32.6
Part-time or temporary work	76	31.3	35	30.7	41	31.8
Full-time permanent position	85	35.0	39	34.2	46	35.6
Family size^a^	2.71	1.66	2.9	1.6	2.5	1.7
Religiosity						
Not highly religious	17	7	4	3.5	13	10.1
Highly religious	226	93	110	96.5	116	89.1
Reasons for immigration						
Involuntary	211	86.8	99	86.8	112	86.8
Voluntary	32	13.2	15	13.2	17	13.2
Family situation						
Waiting to re-unite	10	4.1	6	5.3	4	3.1
Living with core family	233	95.9	108	94.7	125	96.9
Length of stay in Norway						
1–7 years	153	63.0	68	59.7	85	65.9
7–12 years	36	14.8	20	17.5	16	12.4
>12 years	54	22.2	26	22.8	28	21.7

a Reported values are mean (*SD*).

### Acculturation

Acculturation-related characteristics of the study population according to possible 12-month depression are presented in Table 2. None of these characteristics was statistically significantly associated with depression. Almost all (93%) participants reported that they were happy living in Norway. Overall, substantial proportion of the sample self-reported medium to very good Norwegian language skills (94.6%), read Norwegian (60.1%) news at least weekly, were seldom (52.3%) or never (34.2%) visited by Norwegians, and reported to seldom or never have received support from Norwegians (87.3%). Furthermore, over a third of the participants had a feeling of either being denied housing (44.0%) or being refused employment (35.8%) because of their immigrant background. As many as 79.8% reported participating in social activities within the Eritrean community.

**Table 2.** Self-reported Acculturation-related Characteristics of the Study Population by Possible 12-Month Depression According to Major Depression Inventory.

Characteristics	Total (*N* = 243)	No depression (*N* = 202)	Possible depression (*N* = 41)	*p*-Value
	*n* (%)	*n* (%)	*n* (%)	
Happy living in Norway				.930
Yes	226 (93.0)	188 (93.1)	38 (92.7)	
In between	17 (7.0)	14 (6.9)	3 (7.3)	
Norwegian language skill				.466
Very good	69 (28.4)	61 (30.2)	8 (19.5)	
Good	96 (39.5)	80 (39.6)	16 (39.0)	
Medium	65 (26.7)	50 (24.8)	15 (36.6)	
A bit poor	11 (4.5)	9 (4.5)	2 (4.9)	
Poor	2 (0.8)	2 (1.0)	0 (0.0)	
Read Norwegian news (paper or online)				.595
Daily	87 (35.8)	70 (34.7)	17 (41.5)	
Weekly	59 (24.3)	51 (25.2)	8 (19.5)	
Seldom	65 (26.7)	56 (27.7)	9 (22.0)	
Never	32 (13.2)	25 (12.4)	7 (17.1)	
Read Eritrean news (paper or online)				.985
Daily	16 (6.6)	13 (6.4)	3 (7.3)	
Weekly	7 (2.9)	6 (3.0)	1 (2.4)	
Seldom	84 (34.6)	69 (34.2)	15 (36.6)	
Never	136 (56.0)	114 (56.4)	22 (53.7)	
Visited by Norwegians during the last year				.939
Daily	9 (3.7)	8 (4.0)	1 (2.4)	
Weekly	24 (9.9)	19 (9.4)	5 (12.2)	
Seldom	127 (52.3)	106 (52.5)	21 (51.2)	
Never	83 (34.2	69 (34.2)	14 (34.1)	
Received help/support from Norwegians last year				.084
Daily	8 (3.3)	4 (2.0)	4 (9.8)	
Weekly	23 (9.5)	20 (9.9)	3 (7.3)	
Seldom	110 (45.3)	93 (46.0)	17 (41.5)	
Never	102 (42.0)	85 (42.1)	17 (41.5)	
Participated in social activity within Eritrean community, last year				.257
Daily	8 (3.3)	5 (2.5)	3 (7.3)	
Weekly	186 (76.5)	154 (76.2)	32 (78.0)	
Seldom	40 (16.5)	36 (17.8)	4 (9.8)	
Never	9 (3.7)	7 (3.5)	2 (4.9)	
Denied housing (rent or purchase) because of immigrant background				.694
Yes, definitely	12 (4.9)	10 (5.0)	2 (4.9)	
Yes, I have such a feeling	95 (39.1)	79 (39.1)	16 (39.0)	
No, never	34 (14.0)	26 (12.9)	8 (19.5)	
Don’t know	102 (42.0)	87 (43.1)	15 (36.6)	
Refused to employment because of immigrant background				.193
Yes, definitely	9 (3.7)	9 (4.5)	0 (0.0)	
Yes, I have such a feeling	78 (32.1)	62 (30.7)	16 (39.0)	
No, never	38 (15.6)	29 (14.4)	9 (22.0)	
Don’t know	118 (48.6)	102 (50.5)	16 (39.0)	

### Depression, Life Events, and Post-traumatic Symptoms

The prevalence of depression in the recent 2-week period as indicated by the CES-D and MDI were 0.8% and 1.2%, respectively. Possible 12-month depression as indicated by MDI was found positive among 41 (16.9%) participants. Thirty-six (14.8%) participants responded positively to the question ‘Have you felt during the last12-months that life wasn’t worth living?’

Table 3 shows the exposure to adverse life events as assessed by Life events checklist (LEC) by depression status. A large majority (*n* = 205; 84.4%) of the participants reported of having experienced at least one adverse life event. Events happening personally was reported by 195 participants (80.0%), while 56 (23.0%) reported having witnessed such an event. Of the total sample, 46 participants (18.9 %) had a history of at least one personally experienced history and at least one such event witnessed. Among these subgroups, no subgroup showed statistically significant associations with 12-month major depression. Compared to the participants without depression, those with 12-month depression had higher number of events personally experienced (means = 2.06 *SD* 1.59 vs. 2.89 *SD* 2.77; [*t*(231) = −2.593, *p* = .075] albeit statistical significance was at trend level. Similar trend, but weaker difference was observed in case of having witnessed such events. Life events associated with possible depression status were assault with weapon, life-threatening illness or injury, homicide or suicide or unspecified major stressful life event (Table 3).

**Table 3.** Exposure to Adverse Life Events as Assessed by Life Events Checklist (LEC) Among Eritrean Adults Living in Norway by Depression Status.

Characteristic	Total sample (*N* = 243)	No depression (*N* = 202)	Possible depression (*N = *41)	*p-*Value
	*n* (%)	*n* (%)	*n* (%)	
1. Natural disaster	33 (13.6)	25 (12.4)	8 (19.5)	.22
2. Fire or explosion	12 (4.9)	7 (3.5)	5 (24.4)	
3. Road traffic accident	57 (23.5)	48 (23.8)	9 (22.0)	.80
4. Other serious accidents (work, home, or recreational activity)	11 (4.5)	6 (3.0)	5 (12.2)	
5. Exposure to toxic substance	6 (2.5)	3 (1.5)	3 (7.3)	
6. Physical assault (hit, slapped, kicked, beaten up)	66 (27.2)	53 (26.2)	13 (31.7)	.47
7. Assault with a weapon	25 (10.3)	16 (7.9)	9 (22.0)	<.01
8. Sexual assault	0 (0.0)	0 (0.0)	0 (0.0)	
9. Other undesired or unpleasant sexual experience	3 (1.2)	1 (0.5)	2 (4.9)	
10. Combat or exposure to a warzone	138 (56.8)	118 (58.4)	20 (48.8)	.26
11. Captivity (being kidnapped, prisoner of war)	9 (3.7)	6 (3.0)	3 (7.3)	
12. Life-threatening illness or injury	49 (20.2)	35 (17.3)	14 (34.1)	.01
13. Severe human suffering	53 (21.8)	42 (20.8)	11 (26.8)	.39
14. Sudden, violent death (homicide, suicide)	49 (20.2)	35 (17.3)	14 (34.1)	.01
15. Sudden unexpected death of relatives or friends	78 (32.1)	63 (31.2)	15 (36.6)	.5
16. Serious injury or death caused to others	15 (6.2)	12 (6.0)	3 (7.3)	
17. Any other major stressful event or experience	16 (6.6)	9 (4.5)	7 (17.1)	<.01

*Note. p*-values not calculated for comparisons involving cells with ⩽5 subjects.

Factors significantly associated with 12-month depression were older age ([*t*(241) = −3.043, *p* = .0003]), residing in Norway for longer period of time ([*t*(241) = −2.163, *p* = .035]), having recent conflicts in intimate relationship [χ^2^ (2, *n = *243) = 4.8, *p < *.028] and higher number of adverse life events [*t*(241) = −2.906, *p* = .004]. Furthermore, 40 participants had PTSD symptoms (17.1%), and the presence of PTSD symptoms was significantly associated with 12-month depression [χ^2^ (2, *n = *243) = 18.3, *p < *.001]. We did not observe a statistically significant association between 12-month depression and gender, employment status, self-report of living happily in Norway, status of living with core family or awaiting re-union.

Adjusted and unadjusted odds ratios for 12-month depression symptoms according to binary logistic regression analysis are presented in Table 4. The adjusted model consisted of the variables age, sex, and length of stay in Norway, PTSD symptoms, alcohol problems and number of adverse life events that were either self-experienced or witnessed. Of those variables, PTSD symptoms (OR = 4.2 (95% CI: 1.9 to 9.3, *p* < .001) and the number of adverse life events (OR = 1.05 (95% CI: 1.01 to 1.1, *p* < .001) withstood adjustment.

**Table 4.** Odds Ratio with 95% Confidence Interval for Past-Year Depression Symptoms Among the Sample.

Characteristic	Reference category	OR [95% CI]	aOR [95% CI]
Age		1.052 [1.017–1.089]**	1.031 [0.987–1.076]
Sex	Male	1.384 [0.706–2.715]	1.372 [0.654–2.878]
Length of stay in Norway		1.05 [1.01–1.09]*	1.024 [0.971–1.079]
PTSD symptoms	No PTSD	4.747 [2.223–10.135]***	4.164 [1.862–9.310]***
Alcohol problems	No alcohol problems	0.863 [0.312–2.386]	0.732 [0.232–2.317]
Number of adverse life events^a^		2.697 [1.348–5.393]**	1.054 [1.006–1.105]*

*Note. R*^2^ value for the model = .195. PTSD = Post traumatic stress disorder; aOR = adjusted odds ratio.

a Number of adverse life events included both self-experienced and witnessed events.

* *p* < .05, **p < .01, ***p < .001.

## Discussion

This comprehensive investigation of depression and associated factors among adult Eritrean immigrants in Norway revealed that a large majority of the group has experienced adverse life events, and that the number of such exposures is linearly associated with a higher risk of developing depression. We also identified older age, longer duration of residence in Norway, recent conflicts in intimate relationship and presence of PTSD symptoms as relevant correlates of 12-month depression. Analysis indicated that the presence of PTSD symptoms and higher number of adverse life events are potential independent predictors of depression in the sample. The data reflects important aspects of the integration of the study population within the Norwegian society and provides valuable insights into immigrant mental health.

The rates of depressive symptoms were found low both during the past week (0.8%) and the recent 2-week period (1.2%), despite probable depression during these time frames were evaluated separately using the validated and widely used instruments CES-D and MDI respectively. These rates compare to global estimated prevalence of depression among 5% adults (World Health Organization, 2023). Although variations in the prevalence rates reported in the literature may be due to the choice of assessment tools and how depression is operationalised (Moreno-Agostino et al., 2021), these are useful tools to screen clinically significant depression in diverse populations (Schade et al., 1998). On the contrary, the rate of 12-month depression was detected in a much larger subsample (16.9%) than would be expected in the general population. Additionally, it is rather concerning that as many as 15% participants indicated lack of zest about life altogether. Uniformly designed studies using valid, reliable, and comparable methods across countries have shown a large variation of depression rates. For example, the World Health Surveys in 53 countries showed depression rates ranging between 0.4% and 15.7% (Rai et al., 2013). Epidemiological studies with cross-national comparisons and meta-analyses have estimated pooled prevalence at around 5% to 7% for 12-month depression, with some evidence indicating that the rates vary minimally between low-and high-income countries when considered as groups (Bromet et al., 2011; Lim et al., 2018). Nonetheless, the low point prevalence in recent time frames and higher rates in 12-month rates in our sample may have different explanations. One could speculate that, besides other contextual factors, stigma attached to reporting symptoms of depression that is ongoing or in the recent time frame may potentially be stronger than is the case with report of the past. Relatedly, the lifetime prevalence of depression among Ethiopian immigrants in Toronto, Canada was estimated to be just under 10%, three times the rates reported among locals in Ethiopia (Fenta et al., 2004). A study of unaccompanied asylum-seeking Afghani, Somalian, and Iranian youths in Norway found major depressive disorder among 9% (Jakobsen et al., 2014). Studies of refugee population resettled in Europe, Australia, and North America comprising of 14 different samples found a weighted average of 5% prevalence of major depression (Fazel et al., 2005). Thus, the rates of 12-month depression identified in the current study is much higher than generally comparable samples. The rates should also be viewed in the context of significantly higher rates of psychological distress among immigrants in Norway coming from low-income countries compared to those from higher-income countries (Thapa et al., 2007).

Rates of depression and factors contributing to it may differ between refugee populations and native residents (Priebe et al., 2016; Solé-Auró & Crimmins, 2008). Factors often highlighted in the literature concerning depression among African immigrant populations in Europe and North America are younger age, female sex, stigma, experiences of premigration trauma and PTSD, refugee camp internment, and postmigration stressful events including work status, acculturation challenges, and ethnic discrimination (Abebe et al., 2014; Ermansons et al., 2023; Fenta et al., 2004; Missinne & Bracke, 2012). Previous research on the correlates of psychological distress among immigrant populations in Oslo has implied unemployment and recent negative life events as common risk indicators, with men being more vulnerable to past trauma in addition to factors such as lacking close contacts with the natives and experiencing job denials (Thapa & Hauff, 2005). The same study reported that immigrant women had higher risks for distress with increasing age, when living without a partner and when experiencing denial of housing because of immigrant background. In the current sample, the rates did not vary substantially by employment and marital status. In fact, none of the measures of acculturation appeared to be associated with depression in the current sample. Notably, the percentage of individuals reporting no arenas of contact between the immigrants and the native population have halved from 33% in 2002 to 15% in 2025 (Statistics Norway, 2025a). During the same period, attitudes towards immigrants and immigration have become increasingly positive (Statistics Norway, 2025a). We observed that while 93% participants reported that they were happy living in Norway, 14.8% participants felt during the last12-months that life wasn’t worth living. The exact reason underlying this paradoxical finding is unknown, however, this might be related to cultural differences in expressing mental distress, or to different meanings attached to the concept of happiness. It might also be possible that the former finding reflects participants’ satisfaction towards their choice of host nation irrespective of the internalising symptoms of depression.

Although the prevalence of depression was higher among women (19.3%) compared to men (14.7%), the difference was not statistically significant. The results are generally aligned with the findings from immigrant and non-immigrant populations demonstrating higher prevalence among women (Aroian & Norris, 2002; Brendler-Lindqvist et al., 2014; Fenta et al., 2004; Piccinelli & Wilkinson, 2000). Similar to our findings, higher rates of depression have been reported among married women compared to married men (Park et al., 2013) probably owing to gender-specific challenges with acculturation, life events etc. Our data supported increased prevalence of depression with higher age (Molla et al., 2016), but age did not prove to be an independent predictor of depression. We did not find the major impact of employment status, self-report of living happily as immigrant and status of living with core family. Low language skills and feeling of powerlessness are suggested to increase the risk of depression and PTSD (Dalgard, 2008; Nakash et al., 2015). Thus, expected and perceived social support as well as the cultural aspects of the country in which the immigrant has settled may have implications, but the variables that we investigated did not reveal significant correlates representing those dimensions.

Stressful life events are strongly associated with the onset of depression and is demonstrated in immigrant groups in Scandinavia (Glavin et al., 2009; Nakash et al., 2016). Cumulative effects of adverse life events have been documented in large population-based samples (Tibubos et al., 2021). Our study demonstrated that PTSD symptoms and number of adverse life events were frequent, and both factors independently predicted depression. Many immigrants have experienced adverse life events and subjected to post-migration living difficulties, mainly lack of work permits and poverty that together may increase risks for developing PTSD and depression (Aragona et al., 2012; Rodolico et al., 2020).

Length of stay in the resettled country is an important parameter for mental health status among immigrant population. We found a linear association between the number of years one has resided in Norway and the prevalence of depression. The current data is insufficient to explain the underlying reasons for this finding. However, some literature exists on the topic. For example, it has been reported that longer period of stay in Australia was directly related to psychological distress among Iraqi refugees (Brendler-Lindqvist et al., 2014). It is possible that different phases of culture shock, socioeconomic disparities, greater social isolation, and unmet expectations may play a role, while traumatic pre-migration experiences may compound the risk (Abebe et al., 2014; Priebe et al., 2016). Newer challenges including evolving dynamics of the country and society in which one is resettled, and perceived fulfilment of life could be important variables for further investigation. A retrospective study in Denmark showed that referrals, as a proxy for incidence of mental disorder, increased with increased length of stay in asylum centres (Hallas et al., 2007). A major motivation for immigration is the pursuit of a better life; however, when these expectations remain unmet in the host country, individuals may become vulnerable to depression and other mental health issues. A report commissioned by the WHO Regional Office for Europe on Migration highlighted the roles of socioeconomic conditions in increasing depression risks 5 years after resettlement of immigrant groups and that the groups encounter multiple barriers to accessing mental health care (Priebe et al., 2016). The report called for good practices for mental health care including promotion of social integration, improved outreach services, professional training, and information on entitlements and available services as well as training to service providers (Priebe et al., 2016).

The correlates identified in the current analysis should be interpreted with caution because of the possibility of unmeasured confounders and risk of type II error owing to several comparisons consisting of only few subjects in some groups. Furthermore, we used standardised questionnaires to assess depressive symptoms rather than structured interview schedules, such as the Structured Clinical Interview for DSM Disorders (SCID) or the Mini International Neuropsychiatric Interview (MINI), which are designed for diagnosing depression. Although PTSD symptoms were assessed in the sample, another limitation of the current study is that we did not establish diagnoses of relevant psychiatric comorbidities, particularly anxiety disorders and PTSD. The low non-response rate (six individuals) likely had minimal impact on the findings, though refusal to participate may reflect stigma surrounding mental illness. Future studies of immigrant mental health should preferably be conducted on larger samples and account for the differences in reasons for immigration, pre-migration trauma and acculturation stress, family situation, gender and socioeconomic footing as well as duration of resettlement, geographical location, and other post-immigration factors. Longitudinal studies are sorely needed. Future studies should explore how immigrants subjectively experience depressive symptoms and interpret them within the cultural and social context of the host country, especially in relation to their life trajectory. Furthermore, there is growing interest in previously overlooked aspects of the acculturation process, including the impact of how majority-group members adapt to increasingly diverse societies (Kunst et al., 2021). Treating depression among immigrants requires more than trauma-informed care; clinicians should adopt culturally responsive approaches that address migration-related stressors and systemic barriers (Fennig, 2021). Preventive policy-level implications include integration of mental health into immigration and resettlement programmes, investing in culturally and linguistically appropriate mental health services and supporting empowerment through community-led initiatives.

## Conclusion

The present study indicates high prevalence of depression among adult Eritrean immigrants in the Oslo region of Norway, with adverse life events being potentially major contributors to depression. The data presented in this study provide insights into mental health status and reflects important aspects of the integration of adult Eritrean immigrants within the Norwegian society. We believe this study is the hitherto only comprehensive investigation of the depression and associated factors solely within an adult Eritrean immigrant population in a European setting. It should be noted that the data for this study were collected prior to the pandemic of Covid-19. Hence finding of this study could serve as baseline research on the mental health status of adult Eritrean immigrants living in a developed economy setting. It furthers the knowledge about the immigrant mental health issues, challenging many health care systems.
